# Fecal calprotectin as an indicator in risk stratification of pouchitis following ileal pouch–anal anastomosis for ulcerative colitis

**DOI:** 10.1080/07853890.2022.2162115

**Published:** 2023-01-03

**Authors:** Rui-Bin Li, Chun-Qiang Li, Shi-Yao Zhang, Kai-Yu Li, Zhi-Cheng Zhao, Gang Liu

**Affiliations:** Department of General Surgery, Tianjin Medical University General Hospital, Tianjin, China

**Keywords:** Ulcerative colitis, ileal pouch–anal anastomosis, pouchitis, fecal calprotectin, Biomarker, complication

## Abstract

**Background:**

Pouchitis is the most common complication following restorative proctocolectomy and ileal pouch–anal anastomosis (IPAA) for ulcerative colitis (UC). Fecal calprotectin (FC) is a noninvasive indicator of the intestinal inflammatory status. This study was conducted to evaluate the clinical value of the FC concentration for the diagnosis and risk assessment of pouchitis.

**Patients and methods:**

This retrospective study involved patients who underwent IPAA for UC at Tianjin Medical University General Hospital from January 2015 to January 2019. The patients were categorized into pouchitis and non-pouchitis groups based on their Pouchitis Disease Activity Index (PDAI) score. Laboratory indicators, including the FC concentration, were collected from both groups.

**Results:**

Sixty-six patients with UC after IPAA were included in the study and divided into the non-pouchitis group (*n* = 40) and pouchitis group (*n* = 26). The correlation coefficient between the FC concentration and the PDAI score was 0.651 (*p* < 0.001). Receiver operating characteristic analysis showed that the FC cut-off value for predicting pouchitis was 579.60 μg/g (area under the curve, 0.938). The patients were then divided into three subgroups according to their PDAI score (0–2, 3–6, and ≥7), and significant differences in the FC concentration were found among the three subgroups. The best FC cut-off value for predicting a high risk of pouchitis (PDAI score of 3–6) was 143.25 μg/g (area under the curve, 0.876).

**Conclusions:**

FC is a useful biomarker in patients with pouchitis. Patients are advised to regularly undergo FC measurement to monitor for pouchitis. An FC concentration in the range of 143.25–579.60 μg/g is predictive of a high risk for pouchitis, and further examination and preventive treatment are necessary in such patients.

**KEY MESSAGES**
- Fecal calprotectin can be used to quantify pouch inflammation.
- Fecal calprotectin can be used to predict a high risk of pouchitis.

## Introduction

Restorative proctocolectomy with ileal pouch–anal anastomosis (IPAA) is currently the standard surgical procedure for treatment of refractory or recurrent ulcerative colitis (UC) [1]. One of the major postoperative complications of this procedure is pouchitis, a nonspecific inflammatory disease of the ileal pouch, and its cumulative prevalence may reach 50% [2]. The etiology of pouchitis is unclear but may be related to fecal sludge [3], intestinal flora translocation, or genetic susceptibility. The occurrence and prognosis of pouchitis are closely related.

A frequently used diagnostic criterion in clinical practice is the Pouchitis Disease Activity Index (PDAI) [4], and pouchitis is defined as a PDAI score of ≥7. However, the PDAI is invasive, expensive, and time-consuming; has low patient compliance; and cannot facilitate dynamic monitoring of disease. Therefore, accurate assessment of pouchitis using noninvasive biomarkers is needed. Noninvasive biomarkers are being increasingly used in the diagnosis and treatment of inflammatory bowel disease. When intestinal inflammation occurs, the quantity of fecal calprotectin (FC) (a protein found in the cytosol of neutrophils) increases in the feces. Measurement of FC has been demonstrated to be a useful noninvasive tool in the diagnosis of inflammatory bowel disease. Measurement of FC has also recently been investigated in acute pouchitis. A study by Thomas et al. [5] showed that the FC concentration was correlated with the percentage of mature granulocytes and activated macrophages in the lamina propria; thus, the FC concentration can be used to quantify pouch inflammation and help in the diagnosis and evaluation of pouch inflammation. Pakarine et al. [6] found a positive correlation between the FC concentration and pouchitis in pediatric patients with UC (*r* = 0.468, *p* < 0.01); the FC concentration was 71 ± 50 μg/g in children without pouchitis and 290 ± 131 μg/g in those with pouchitis. Johnson et al. [7] evaluated 46 postoperative patients with UC who underwent endoscopy. Of these 46 patients, 6 were diagnosed with pouchitis and pre-pouch ileitis, 13 with pouchitis alone, and 27 without pouchitis, and their median FC concentration was 865, 145 and 56 μg/g, respectively; additionally, the FC concentration was correlated with the PDAI score [7]. However, the potential role of FC in evaluating the severity of pouchitis has not been explored. Therefore, this study was performed to assess the clinical value of FC in the diagnosis and risk assessment of pouchitis.

## Patients and methods

Patients with UC who underwent IPAA in Tianjin Medical University General Hospital from January 2015 to January 2019 were selected for this study. The inclusion criteria were a diagnosis of histologically confirmed UC, closure ileostomy following IPAA for UC, and an age of 16–70 years. The exclusion criteria were treatment with antibiotics, immunomodulators, steroids, or nonsteroidal anti-inflammatory drugs 1 month prior to review and the development of other pouch-related complications (e.g. anastomotic leakage, pelvic abscess, pouch fistula, or anastomotic stricture). Temporary endoscopy was performed in all patients for follow-up purposes. Meanwhile, laboratory indices (including routine blood indices, C-reactive protein (CRP) concentration, erythrocyte sedimentation rate (ESR), and FC concentration) were obtained. Patient grouping was based on the PDAI scoring criteria; i.e. pouchitis was defined as a PDAI score of ≥7, and non-pouchitis was defined as a PDAI score of <7. All statistical analyses were performed using SPSS version 25 software (IBM Corp., Armonk, NY, USA). Student’s *t*-test was used for univariate analysis. Associations of the FC concentration with the blood test results and PDAI score were evaluated with Spearman’s rank sum test. A receiver operating characteristic (ROC) curve was drawn to estimate the area under the curve (AUC) and the best cut-off levels of the FC for risk stratification. In each analysis, a *p* value of <0.05 was considered statistically significant. This study was performed in accordance with the Helsinki Declaration of Human Rights and was approved by the institutional review board of Tianjin Medical University General Hospital. Written informed consent was obtained from all of the patients.

## Results

### Patients’ baseline demographic characteristics

The patients’ baseline characteristics are shown in Table 1. Of the 66 patients included in this study, 26 were diagnosed with pouchitis, with a median PDAI score of 10.00 (interquartile range, 9.00–11.25). The median FC concentration in patients with and without pouchitis was 765.00 μg/g (597.55–963.70 μg/g) and 203.50 μg/g (136.90–450.23 μg/g), respectively (*p* = 0.001). There was a significant difference in the CRP concentration (*p* < 0.001) and ESR (*p* = 0.028) between the two groups.

**Table 1.** Patients’ baseline demographic variables.

	Pouchitis group	Non-pouchitis group	*p*
N	26	40	
Gender(M/F)	10/16	18/22	0.599
Age	44.00 (42.25–51.00)	41.50（36.25–43.75）	0.010
WBC (*10^9^/L)	6.47 (5.64–7.84)	6.15 (5.39–6.84)	0.091
NEU (%)	59.40 (51.90–69.98)	57.2 (52.23–63.78)	0.198
CRP (mg/dl)	0.88 (0.72–1.17)	0.45 (0.27–0.60)	<0.001
ESR (mm/h)	23.00 (18.75–30.50)	13.50 (9.00–26.25)	0.028
FC (ug/g)	764.00 (597.55–963.70)	203.50 (136.90–450.23)	0.001
PDAI score	10.00 (9.00–11.25)	4.00 (3.00–4.75)	<0.001
Symptom score	3 (3.00–4.00)	2.00 (1.00–2.00)	<0.001
Endoscopy score	4 (3.00–4.00)	1.00 (0.00–2.00)	<0.001
Histology score	3 (2.00–4.00)	0.00 (0.00–1.00)	<0.001

Data are presented as n or median (interquartile range).

M: male; F: female; WBC: white blood cell; NEU: neutrophil; CRP: C-reactive protein; ESR: erythrocyte sedimentation rate; FC: fecal calprotectin; PDAI: Pouchitis Disease Activity Index.

### Association between FC concentration and blood test results

The FC concentration was correlated with the CRP concentration (Spearman’s rank correlation coefficient *r* = 0.521, *p* = 0.002) and ESR (*r* = 0.354, *p* = 0.043) but not with the white blood cell (WBC) count (*r* = 0.117, *p* = 0.517) or neutrophil percentage (*r* = 0.009, *p* = 0.962).

### Association between FC concentration and PDAI score

**Figure 1.**
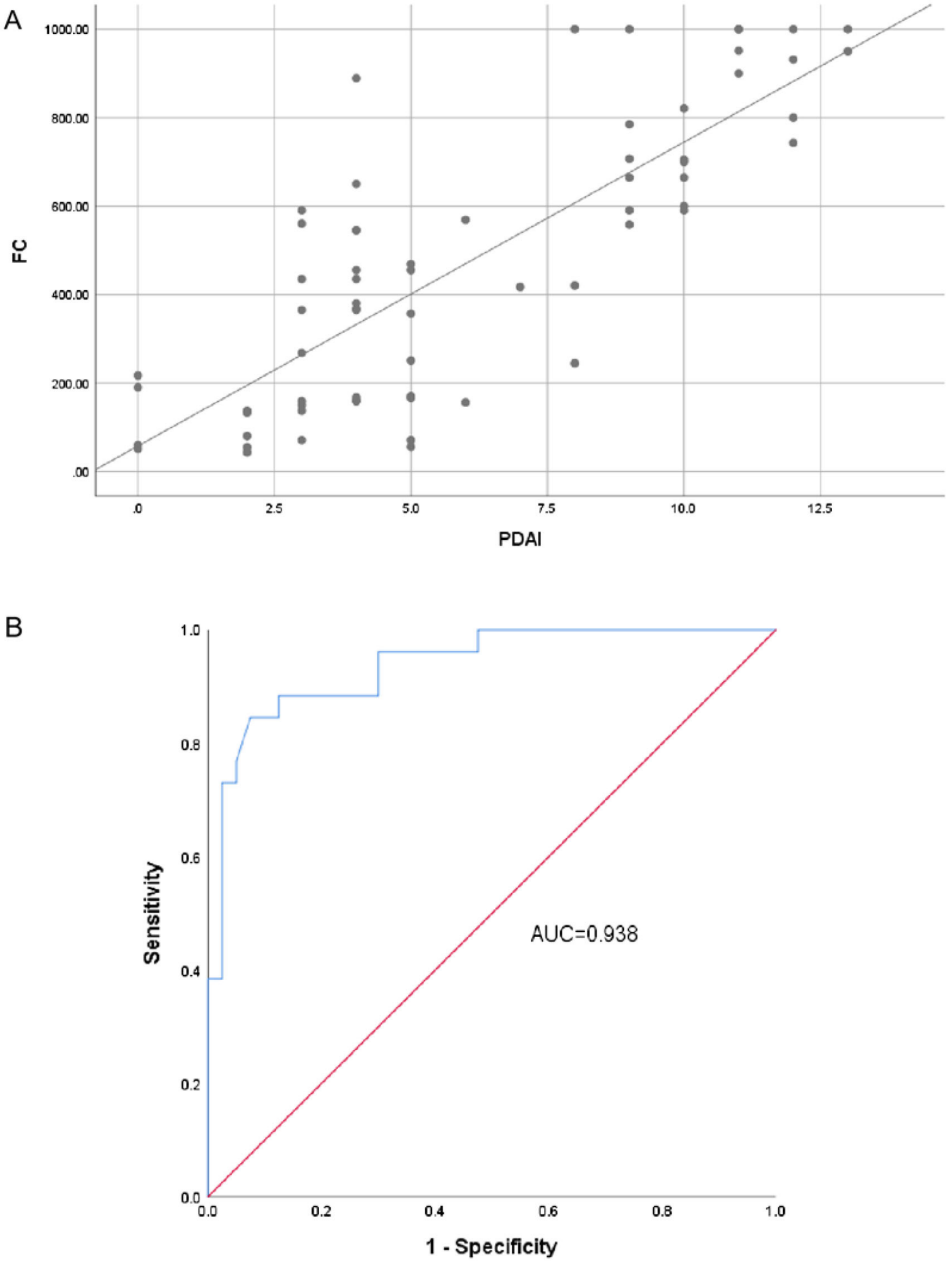
Association between FC concentration and PDAI score. (A) Correlation between PDAI score and FC concentration. (B) Receiver operating characteristic curve of FC concentration for prediction of pouchitis. FC: fecal calprotectin; PDAI: Pouchitis Disease Activity Index; AUC: area under the curve.

The correlation coefficient (Figure 1(A)) between the FC concentration and PDAI score, endoscopy score, and histology score was 0.651 (*p* < 0.001), 0.791 (*p* < 0.001), and 0.696 (*p* < 0.001), respectively. Although the correlation coefficient between the FC concentration and symptom score was relatively low (*r* = 0.406), it was statistically significant (*p* = 0.019).

The best FC cut-off value for predicting pouchitis was 579.60 μg/g (AUC, 0.938) with a sensitivity of 84.6% and a specificity of 92.5% (Figure 1(B)).

### Association between FC concentration and PDAI score in non-pouchitis group

**Figure 2.**
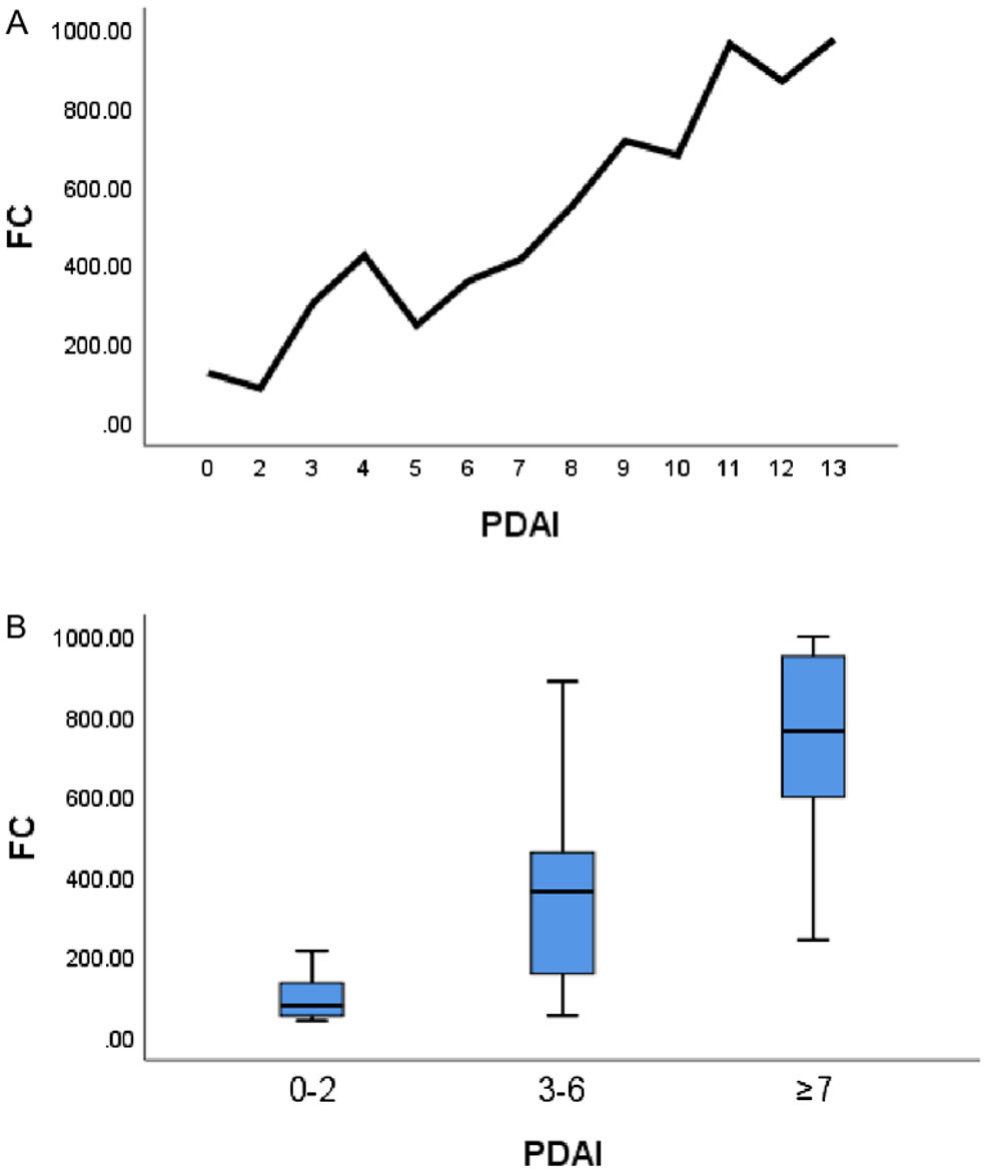
Association between FC concentration and subgroups. (A) Line graph of PDAI scores versus corresponding FC concentrations in non-pouchitis group. (B) The three groups showed statistically significant differences among one another. FC: fecal calprotectin; PDAI: Pouchitis Disease Activity Index.

Among all 66 patients, the range of PDAI scores was 0–13, and the median FC concentration corresponding to each point was calculated to make a line graph of PDAI scores versus the corresponding FC concentrations (Figure 2(A)). The line graph showed a significant difference in the FC concentration between scores of 0–2 and 3–6 in the PDAI score range of 0 to <7. Thus, we divided the PDAI score into three groups: 0–2, 3–6, and ≥7.

The median FC concentration in the 0–2 and 3–6 subgroups was 80.00 μg/g (52.55–163.45 μg/g) and 365.00 μg/g (158.90–469.00 μg/g), respectively. There were significant differences in the FC concentration among the three groups (Figure 2(B)). Thus, we defined patients with a PDAI score of 3–6 as those at high risk for pouchitis.

**Figure 3.**
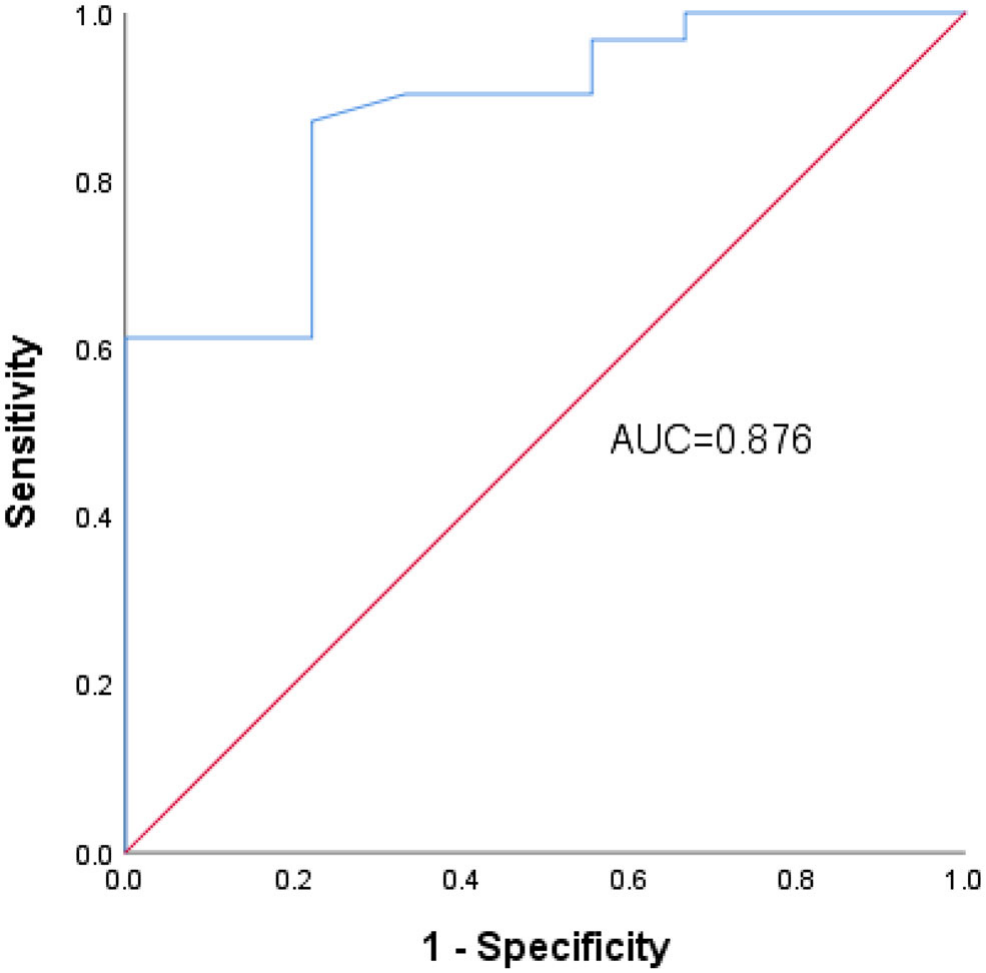
Receiver operating characteristic curve of fecal calprotectin concentration for prediction of pouchitis. AUC: area under the curve.

Based on these results, further ROC analysis was performed for the 0–2 and 3–6 subgroups. The best FC cut-off value for predicting a high risk of pouchitis was 143.25 μg/g (AUC, 0.876) with a sensitivity of 87.1% and a specificity of 77.8% (Figure 3).

## Discussion

Pouchitis is a nonspecific inflammatory disease of the ileal pouch. The main diseases that require IPAA are familial adenomatous polyposis and UC [8]. Patients with UC are more likely to develop pouchitis than are patients with familial adenomatous polyposis [9], and the reason may be related to the abnormal autoimmune status of patients with UC. The main inflammatory indicators reflecting the inflammatory status in patients with UC include the WBC count, CRP concentration, ESR, and FC concentration [10,11]. The blood inflammatory indicators in this study included the WBC count, neutrophil percentage, CRP concentration, and ESR. It is evident that the CRP concentration and ESR are more valuable than the WBC count. Matalon et al. [12] evaluated 71 patients with UC who developed pouchitis and found that the CRP concentration was significantly correlated with the PDAI score (*r* = 0.584, *p* < 0.001). Lu et al. [13] obtained a similar result.

To date, studies on the application of FC measurement in patients with UC have mainly focused on the patients’ preoperative status, including the assessment of disease activity and the effect of drug treatment [14,15]. Few studies have focused on the postoperative status of patients with UC. Research has shown a significant correlation between the FC concentration and pouchitis [16,17]. The present study was performed to evaluate the value of the FC concentration in predicting pouchitis after surgical treatment of UC. The results of this study showed that the FC concentration was significantly higher in patients with than without pouchitis. The correlation coefficient was 0.651 (*p* < 0.001) between the FC concentration and PDAI score. The ROC analysis showed that the best FC cut-off value for predicting pouchitis was 579.60 μg/g (AUC, 0.938) with a sensitivity of 84.6% and a specificity of 92.5%.

Estimation of FC can facilitate identification of low-grade inflammation before it manifests endoscopically or clinically. Inflammation can be seen endoscopically in 30% and histologically in 50% of adults with UC who are in clinical remission [18,19]. Therefore, we evaluated the clinical value of FC in the risk stratification of pouchitis. We drew a line graph to view the trend of the FC concentration in the non-pouchitis group and divided all patients into three subgroups according to the PDAI score: 0–2, 3–6 and ≥7. There were significant differences in the FC concentration among the three subgroups. The best FC cut-off value for predicting a high risk of pouchitis was 143.25 μg/g.

This study had some limitations, including the lack of information about longitudinal clinical end points (e.g. the prognostic value of the FC concentration) and the small sample size of patients with UC. Large-sample and prospective studies are still required.

## Conclusions

The FC concentration is a useful biomarker in patients with pouchitis. Patients are advised to regularly undergo FC measurement to monitor for pouchitis. An FC concentration of 143.25–579.60 μg/g is predictive of a high risk of pouchitis, and further examination and preventive treatment are necessary in such patients.

We acknowledge Angela Morben, DVM, ELS, of Liwen Bianji (Edanz) (www.liwenbianji.cn), who edited the English text of an early version of this manuscript. No funding was received.

## Data Availability

The data that support the findings of this study are available on request from the corresponding author.
